# Investigating persistent measles dynamics in Niger and associations with rainfall

**DOI:** 10.1098/rsif.2020.0480

**Published:** 2020-08-26

**Authors:** Alexandre Blake, Ali Djibo, Ousmane Guindo, Nita Bharti

**Affiliations:** 1 Biology Department, Center for Infectious Disease Dynamics, Penn State University, University Park, PA, USA; 2 Abdou Moumouni University, Niamey, Niger; 3 Department of Research, Epicentre, Maradi, Niger

**Keywords:** measles, seasonal migration, vaccination, metapopulation, sub-Saharan Africa

## Abstract

Measles is a major cause of child mortality in sub-Saharan Africa. Current immunization strategies achieve low coverage in areas where transmission drivers differ substantially from those in high-income countries. A better understanding of measles transmission in areas with measles persistence will increase vaccination coverage and reduce ongoing transmission. We analysed weekly reported measles cases at the district level in Niger from 1995 to 2004 to identify underlying transmission mechanisms. We identified dominant periodicities and the associated spatial clustering patterns. We also investigated associations between reported measles cases and environmental drivers associated with human activities, particularly rainfall. The annual and 2–3-year periodicities dominated the reporting data spectrum. The annual periodicity was strong with contiguous spatial clustering, consistent with the latitudinal gradient of population density, and stable over time. The 2–3-year periodicities were weaker, unstable over time and had spatially fragmented clustering. The rainy season was associated with a lower risk of measles case reporting. The annual periodicity likely reflects seasonal agricultural labour migration, whereas the 2–3-year periodicity potentially results from multiple mechanisms such as reintroductions and vaccine coverage heterogeneity. Our findings suggest that improving vaccine coverage in seasonally mobile populations could reduce strong measles seasonality in Niger and across similar settings.

## 1. Introduction

Measles is one of the most infectious human diseases. With a basic reproduction number between 12 and 18 [1], it was the source of a significant disease burden worldwide in the pre-vaccination era. Despite occasional outbreaks, measles is now largely controlled in high-income countries. However, it remains a major cause of child mortality in sub-Saharan African countries owing to large outbreaks in areas with suboptimal vaccine coverage [2] and caused about 62 000 deaths in this region in 2017 (89% were children under 5 years) [3,4]. The case–fatality ratio is around 5–10% in this region, compared with 1 death in 1000 children in high-income countries, and can reach 20–30% in emergency settings [2].

Measles transmission dynamics have been studied in high-income countries in the pre-vaccination era. This has led to the description of some important threshold and transmission characteristics, including the critical community size [5], the rescue effect owing to population movement and metapopulation connectivity [6,7], and also term-time forcing due to the aggregation of children in schools [8,9]. Some of these characteristics are influenced by environmental constraints such as transport infrastructure, roads and railways because of how they shape population movement, or more broadly human activities [10–12]. These constraints can indirectly support regional persistence, but their nature and the magnitude of their influence can vary substantially across the globe [13].

Measles infection or successful vaccination leads to lifelong immunity. Outbreaks are then the result of the building of the susceptible population through births, but sound immunization strategies can play a major role in preventing cases if the coverage is consistently high enough [4]. The current immunization strategies rely on routine vaccination for at least one dose with or without regular supplementary immunization activities. These strategies succeed in achieving high vaccine coverage in high-income countries but have more nuanced results in low-income regions, including sub-Saharan Africa [14]. These immunization strategies might simply not be optimal for low-income countries [15], where the health system may be weaker, where the environmental constraints differ and where transmission might follow different patterns from those seen in high-income countries. When we see a seasonal pattern in measles cases in a sub-Saharan country, it is linked to human activities other than school terms [16,17], as is demonstrated by a median age of infection below school age [18]. Furthermore, the transport network relies heavily on roads in this region, and the road network is sparser than in other regions in the world [19]. This imposes very different constraints on population movement. In addition, suboptimal vaccine coverage [20] with spatial and temporal heterogeneity modifies measles dynamics in unpredictable ways.

The West African nation of Niger reported high measles mortality in recent years [21] and presents strong seasonality, similar to classical England and Wales measles dynamics, though in Niger the seasonal forcing is due to agricultural activity [16,17,22]. Additionally, the connectivity of the population in Niger with that of countries with suboptimal vaccination coverage, such as Nigeria [23], provides potential avenues of measles spread. The mobility and mixing of infected and susceptible populations can dramatically influence local and regional measles transmission. This could lead to chaotic transmission dynamics locally because of recurrent and unpredictable reintroductions, but it would also support regional disease persistence [24]. Furthermore, the geography of Niger, with dominant steppe and desert in the north, creates a strong population density gradient and drives measles transmission patterns across space. A better understanding of measles dynamics in this setting is necessary to adapt immunization strategies efficiently and address ongoing measles transmission.

**Figure 1.**
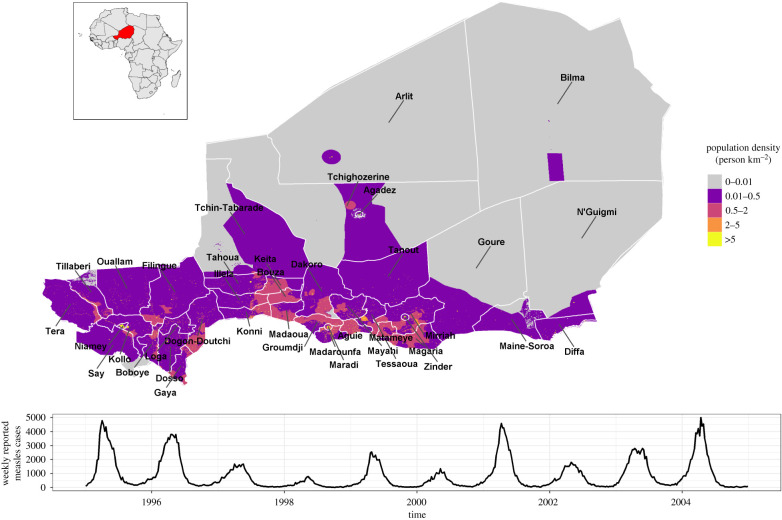
District boundaries in Niger, spatial distribution of population density, and weekly reported measles cases from 1995 to 2004 at country level.

We investigated measles dynamics in Niger at the district level (figure 1) from 1995 to 2004 to identify underlying transmission mechanisms. We also looked for associations between reported measles cases and environmental drivers linked to human activities, including agriculture, a dominant economic activity in Niger, and particularly rainfall and temperature. This could provide valuable insights on how to tailor interventions in this setting with limited resources for maximum effectiveness.

## 2. Methods

We analysed weekly reported measles cases in Niger from 1995 to 2004 at the district level (figure 1), collected by the Ministry of Health of Niger. We investigated periodicities through district power spectra and calculated phase shifts to study synchrony by wavelet analysis with the Morlet wavelet [25]. We systematically considered Niamey, the most populated city, as a reference to estimate the phase shifts [6]. The wavelet analysis allows us to estimate local wavelet power spectra (WPS). WPS reflect the intensity of a range of periodicities and how they vary over time. Averaging the local WPS over the whole 10-year period of the data leads to the global WPS (GWPS). Although temporal variation is lost, the GWPS provide a global picture of the important periodicities in the data. We performed permutation tests (with 1000 permutations) to check if the intensity of the range of periods we considered was significantly higher than could be expected by chance. The GWPS informed us on the periodicities of interest for further investigation, although no periodicity longer than 5 years was explored because of the limited number of observable cycles in our data. We performed a hierarchical clustering (HC) analysis, using an average phase coherency-based distance matrix, to look for spatial patterns for the periodicities of interest. The phase coherency measures the correlation between two spectra for a specific band of interest at a specific point in time [25]. We guided our choice of the number of clusters with the elbow [26] and silhouette plots [27] and the gap statistic [28]. We further investigated the potential impact of changing the number of clusters on the spatial pattern (electronic supplementary material, figures S12 and S13). We calculated the cophenetic correlation between all the clustering trees obtained with the weekly phase coherency for the periodicities of interest to check the temporal stability of the observed pattern. Cophenetic correlation measures the correlation between clustering trees [29]. The more similar the pairwise distances are between two clustering trees the closer to 1 the cophenetic correlation is. Rather than using the average phase coherency to calculate the distance matrix to create the clustering trees, we calculated distance matrices for every week using the phase coherency value of every week. A clustering tree was created for every week, and the cophenetic correlation was calculated for every pair of weekly clustering trees. If the observed clustering pattern is consistent over time, it will produce a high cophenetic correlation between the trees from one week to another. Colour coding the cophenetic correlation between every pair of weekly clustering trees will provide a visual way to observe shifts or temporal stability of clustering patterns for periodicities of interest. Finally, we looked for hierarchy in the spread of the waves of incidence across space. If source–sink metapopulation dynamics consistently trigger some districts and spread measles cases to others, we should observe some consistent delay with positive phase shifts for the sinks using the sources as references. We performed this analysis as follows: we first identified the districts with the lowest median phase shifts (using Niamey as a reference) for one periodicity of interest. We then grouped the remaining districts by spatial adjacency, and recalculated the phase shifts block by block, this time using the district identified with the lowest median shift as a reference. The districts with the lowest median phase shift were considered as potential ‘sources' from which outbreaks spread to the other districts in their blocks, the potential ‘sinks'. Assuming distance-based population movement from source districts, we used the shortest distance between the population-weighted centroids of districts to delineate each block. Population data were collected from the 2010 WorldPop estimates [30]. Earlier estimates were not available, but no large-scale population displacements were reported between our study period and 2010 in Niger. Several cut-offs were used to select the districts with the lowest median phase shifts to explore patterns of hierarchy (electronic supplementary material, figure S9).

We explored the impact of the environmental drivers of agricultural activity, particularly the rainy season, on measles transmission. We interpolated the weekly cumulative rainfall and average temperature from 1995 to 2004 over a grid with cells of 15 km by 15 km after normal score transformation using spatio-temporal kriging on measures collected by weather stations in Niger and within a 300 km surrounding buffer area [31]. These data are publicly available on the National Oceanic and Atmospheric Administration (NOAA) website (https://www7.ncdc.noaa.gov). For every district, we considered the interpolated measures of weekly cumulative rainfall and weekly average temperature. We used administrative boundaries available on the Global Administrative Areas database (GADM) (https://gadm.org). Agadez, Maradi and Zinder cities reported measles cases separately from the rest of their districts, so their boundaries were redrawn using the population density variation across space in the WorldPop data [30] with a buffer area of 7.5 km. The 7.5 km buffer area ensured that at least one point of the grid of interpolated values of rainfall and temperature fell in these areas. The interpolated data were then averaged over the districts. Associations between weekly reported measles cases, rainy season and average weekly temperature were considered with a lagged effect to account for travel time and downstream behavioral impacts. We investigated this association using quasi-Poisson regression with distributed lag models considering a lag as far back as three weeks in the past. We also calculated the cumulative number of cases in the last 4 years as an immune term and the lagged residuals as an autocorrelation term to take into account the specificities of infectious disease transmission [32,33]. Separately, we fit a model to each district that included the rainy season and weekly average temperature and their lags systematically as independent variables. Let *Y_t_*, the number of measles cases reported weekly, be a random variable such that

$$E(Y_{t})=\mu ,$$

$$\text{Var}\;(Y_{t})=\theta \mu_{t}$$

$$\begin{array}{rl} \text{and}\;\log(\mu_{t}) & =\beta_{0}+f\;(\text{wee}{\text{k}}_{t})+\sum_{i=t-3}^{i=t}\;f\;({\text{temp}}_{i})\\ & +\sum_{i=t-3}^{i=t}\beta_{\mathrm{rain}\;i}\text{rai}{\text{n}}_{i}+\beta_{\mathrm{AC}}\text{AC}+\beta_{\mathrm{IT}}\text{IT}+\log(pop_{t}). \end{array}$$

We included the logged estimated population, with yearly estimates, as an offset. The strong seasonality in the data was incorporated through cubic splines, *f*(week*_t_*), with three knots per year. The autocorrelation term, AC, is a lagged residual [33]. The immune term, IT, helps reflect the level of immunity in the population. Without serological data, other proxies can be used and, in our case, the IT is a moving sum of the number of cases in 4 years prior to the week of interest [33]. Various alternative AC and IT have been tested. For the AC, we considered 1-week lagged residuals, a logged and non-logged moving sum of the cases in the 52 weeks prior to the week of interest. Because measles burden is heavier among children under 5 years in Niger and is a lifelong immunizing disease, we used the sum of the number of cases in a moving window of 3, 4, 5 and 6 years. We used a moving window of 4 years as IT and the 1-week lagged residual as AC in the final model because they provided comparable results to the other alternatives but minimized the temporal autocorrelation in the residuals (electronic supplementary material, figure S12). Average weekly temperature was included using cubic splines (*f*(temp_i_)), with the minimum value as reference, considering as far as three weeks of lag. We fitted Fourier terms to the weekly cumulative rainfall with three frequencies to define when the rainy season began and ended in every district, emphasizing the trend more than the actual cumulative rainfall values (electronic supplementary material, figure S7). We also considered as far as three weeks of lag for the effect of the rainy season. We checked for remaining autocorrelation in the residuals of every model (electronic supplementary material, figure S11). The effect of the temperature and the rainy season at different lags and the overall effect were estimated using constrained distributed lag models [32,34]. The association between the measles weekly reported cases and the predictors was measured using a relative risk adjusted on all the predictors (aRR). The significance of the overdispersion was tested [35] and their estimates based on the final quasi-Poisson models are available in the electronic supplementary material, table S1.

All analyses were performed using R v. 3.5.6.

## 3. Results

**Figure 2.**
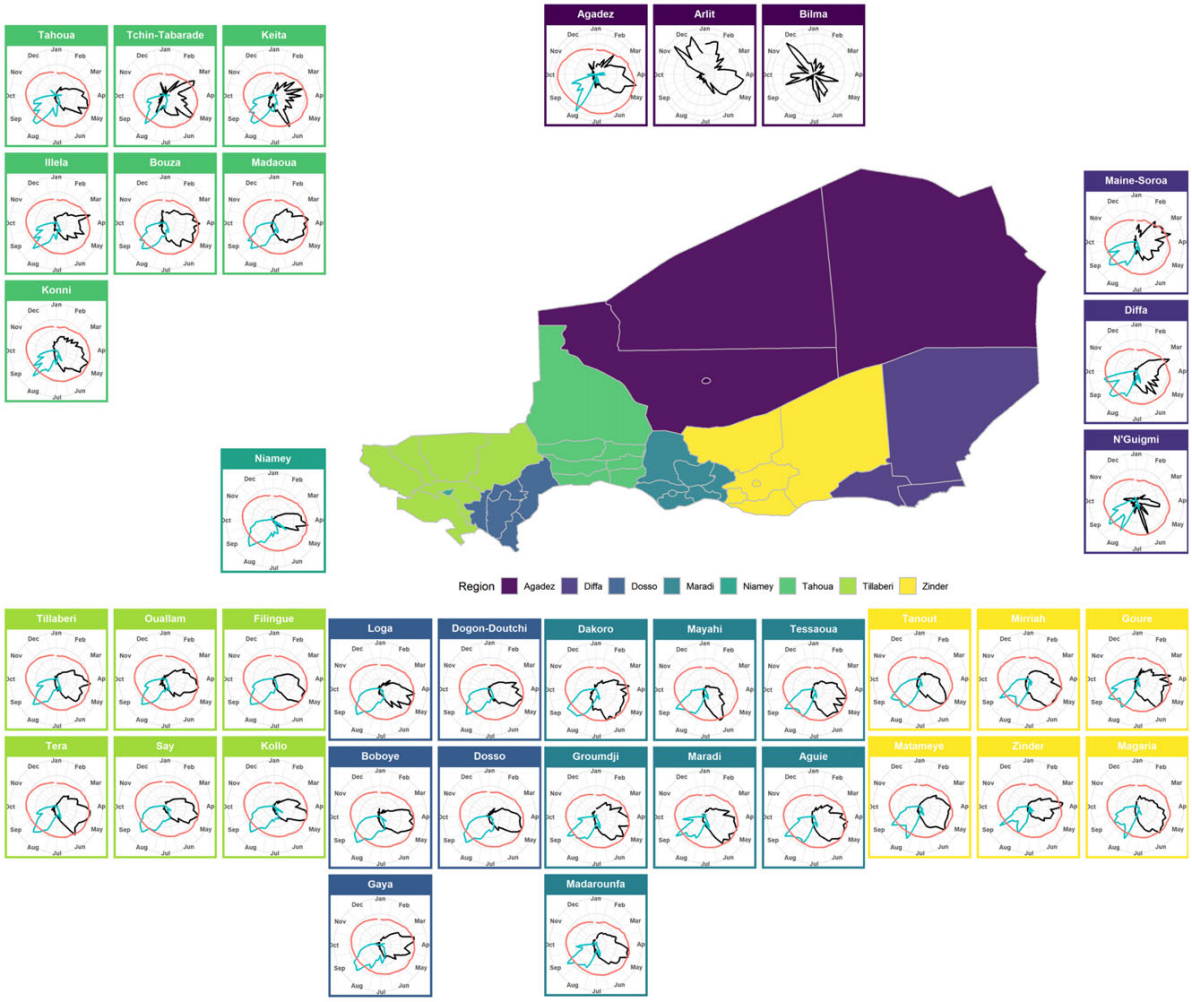
Scaled average weekly reported measles cases (black), weekly cumulative rainfall (blue) and weekly temperature (red) with polar coordinates at district level from 1995 to 2004, Niger. Regions are colour coded, and each cell is colour coded based on the region it belongs to. Every cell uses polar coordinates to highlight potential seasonal patterns. Weekly cumulative rainfall and average weekly temperature have not been interpolated for Bilma and Arlit.

Overall, Niger reported a measles outbreak every year from 1995 to 2004, but the amplitude varied substantially with major outbreaks at the country level in 1995, 1996, 2001 and 2004. The 38 districts displayed variable measles dynamics during this period (electronic supplementary material, Supplementary_Figure_epicurves). The dynamics ranged from what was reported in Niamey, the densely populated capital city, with the largest outbreaks, to the sparsely populated desert areas of Bilma and N'Guigmi, which barely reported any cases. Overall, very strong seasonality was observed (figure 2).

**Figure 3.**
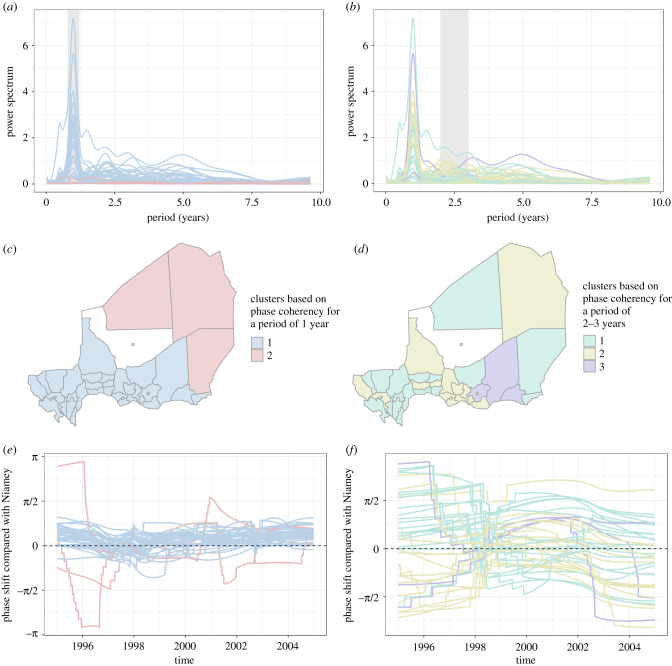
Global wavelet power spectra of the weekly reported measles cases at district level (*a*,*b*), resulting clustering based on average cophenetic correlation (*c*,*d*) and phase shift compared with Niamey (*e*,*f*) for the annual and 2–3-year periodicities from 1995 to 2004 in Niger. The global wavelet power spectra are similar in (*a*) and (*b*), but spectra are coloured based on the cluster the districts belong to in (*c*) and (*d*), and the grey area highlights the bands of interest for all the subfigures below, 0.8–1.2 years and 2–3 years, respectively. The maps in (*c*) and (*d*) are colour coded based on the hierarchical clustering done using the average phase coherency considering the bands highlighted in (*a*) and (*b*), respectively. The subfigures in (*e*) and (*f*) display the phase shifts between every district and Niamey for the periodicities using the bands highlighted in (*a*) and (*b*), respectively. Every phase shift is colour coded based on which cluster the districts belong to in (*c*) and (*d*).

The wavelet analysis revealed a variety of periodicities across districts (electronic supplementary material, Supplementary_Figure_power_spectrum_1 and Supplementary_Figure_power_spectrum_2), as shown in the GWPS (figure 3*a*,*b*). Despite this diversity, there was a strong annual periodicity consistent across districts. In most districts, this annual periodicity was the strongest observed in their respective spectrum. A second broader part of the spectra showed a secondary periodicity varying around 2–3 years, though it was inconsistent. One, two, three or even no peak in the GWPS could be seen in this range of periods. Both the annual and the 2–3 year periodicities were significant (electronic supplementary material, figure S8). We then further investigated the two dominant periodicities using bands of 0.8–1.2 years and 2–3 years.

Using the average phase coherency distance-based matrix of the dominant periodicities, the HC revealed very different spatial patterns for the two periodicities (figure 3). Regarding the annual periodicity, two clusters of contiguous districts were identified. The two clusters split the country along population density and topography. Most of the population lives in the south of the country, and was included in cluster 1 (in pale blue in figure 3*c*). The second cluster included districts with very low population density with desert and steppe, in pale red in figure 3*c*. The clustering pattern was more heterogeneous for the 2–3 years periodicity. Except for cluster 3, the districts of the same clusters were partially scattered, and did not necessarily match population density or clear geographical features (figure 3*d*).

The phase shift between the districts of the first cluster and Niamey had globally little variation for the annual periodicity (figure 3*e* and *f*) and remained mostly between −π/4 and π/4. This constant phase shift suggested synchrony between the districts in the cluster. The three districts from the second cluster displayed large fluctuations over time for the same periodicity, illustrating a very different dynamic in these areas. The phase shifts for the second periodicity were more erratic. They varied over time with a larger magnitude, showing no synchrony overall.

**Figure 4.**
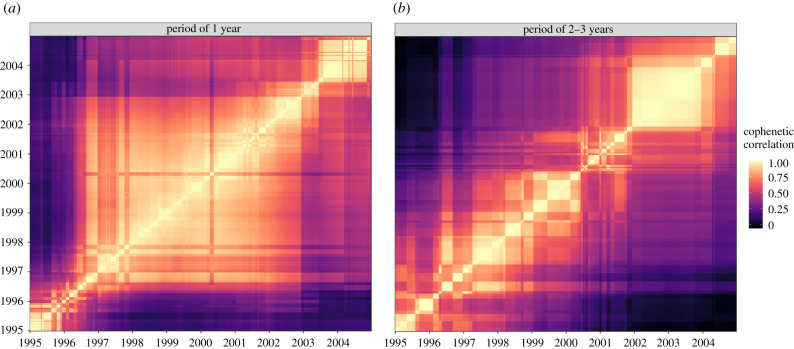
Cophenetic correlation matrices between the clustering trees based on the weekly phase coherency between districts for a period of 0.8–1.2 years and 2–3 years from 1995 to 2004, Niger. Clustering trees were creating using the weekly values of the phase coherency for the periodicities of 0.8–1.2 years and 2–3 years. Cophenetic correlation between every pair of weekly clustering trees and colour coded, with higher cophenetic correlation being lighter. The resulting matrices are organized chronologically to visually display whether the clustering patterns changed over time or remained stable.

The cophenetic correlation matrices calculated using the dendrograms based on the weekly phase coherencies for both periodicities also displayed very different patterns (figure 4). What was previously observed based on the average coherency hid some variation over time. The clustering pattern for the annual periodicity showed essentially three different periods: from 1995 to late 1996, from late 1996 to late 2002, and from late 2002 to 2004. Besides the first period, the cophenetic correlation supported the overall temporal consistency of the clustering pattern for the annual periodicity, at least for about 6 years out of 10. Conversely, the cophenetic correlation for the second periodicity showed a clustering pattern constantly drifting over time. The clustering observed for the second periodicity with the average phase coherency hid a collection of patterns through which it drifted over time.

**Figure 5.**
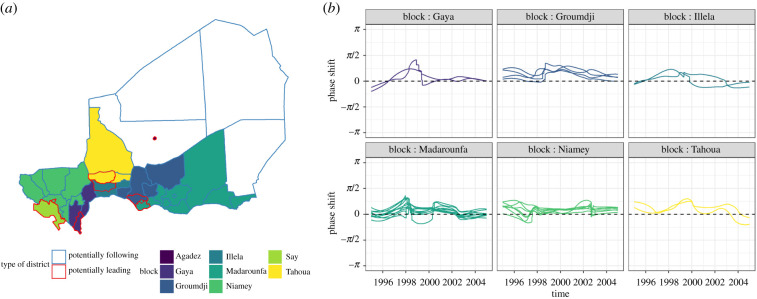
Map of the blocks grouping potential source and potential sink districts based on the distance between population-weighted centroids (*a*) and phase shifts between the potential sink districts and the closest potential source district* (*b*) considering the annual periodicity in reported measles cases from 1995 to 2004 in Niger. The districts with red borders in (*a*) were identified as potentially leading because of their lowest phase shifts for the annual periodicity, whereas the blue districts were identified as potentially following. *: Agadez and Say are not represented in (*b*) because they are potential source districts with no potential sink district.

The median phase shifts of the annual periodicity, with Niamey as a reference, reflected a bigger lag in areas with lower population density with moderate negative correlation between the median phase shift and the logged mean population density (*c* = −0.42, *p* = 0.011). When considering eight ‘potential source districts', six had other ‘potential sink districts' in their blocks. Within their respective blocks, the ‘potential source districts' displayed mostly positive small phase shifts, although not always consistently over time (figure 5). The phase shifts in every block often showed a different pattern from the beginning until about late 1996, from late 1996 until late 2002, and from late 2002 until the end of 2004. This variation over time was consistent with figure 4. Although there was some variation, similar observations could be made when we considered different numbers of potential sources (electronic supplementary material, figure S9). Similar patterns were not observable when considering the second periodicity and supported hierarchy only anecdotally in this case (electronic supplementary material, figure S8).

**Figure 6.**
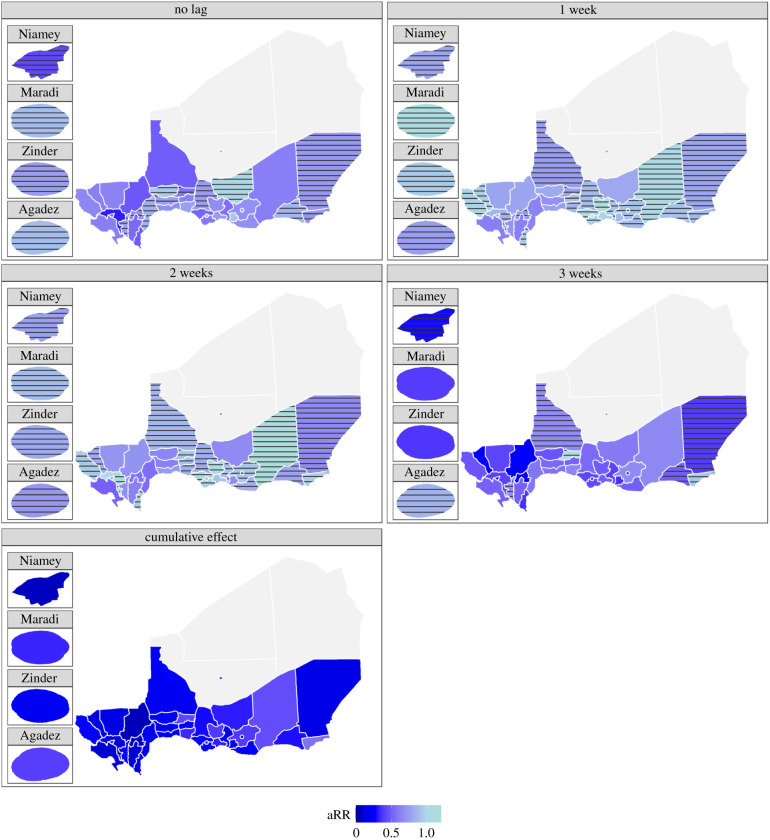
Adjusted relative risk (aRR) of the association between the rainy season and measles incidence with zero to three weeks lags and the cumulative effect from 1995 to 2004 in Niger. The district has horizontal stripes if the confidence interval includes 1. Niamey, Maradi, Zinder and Agadez are also added in insets to make the interpretation of those small areas easier. The value of the aRR is colour coded with darker blue for values closer to 0, meaning a lower risk of reporting measles cases associated with this lagged effect of the occurrence of the rainy season.

Regarding the association with environmental variables, particularly the rainy season, Bilma and Arlit were excluded from this analysis, because they reported too few cases to perform the analysis and displayed a very different dynamic from the southern part of the country. In every district, the overdispersion parameter was significantly different from 1 or close to significance. These estimates are available in the electronic supplementary material. Most of the aRR showed a negative association between reporting measles cases and the rainy season (figure 6). Not every lag showed a significant association, but the cumulative effect over three weeks was systematically significant (electronic supplementary material, figure S10). Little to no autocorrelation remained in the residuals of our models (electronic supplementary material, figure S11).

## 4. Discussion

We identified two main periodicities in the measles dynamics in Niger from 1995 to 2004: a strong, consistent and stable annual periodicity and a weaker, inconsistent and unstable 2–3-year periodicity. In addition, reported measles cases showed a significant association with rainfall and temperature with various lags. This dual periodicity illustrates different transmission mechanisms and their relative impact on measles dynamics. The association with the rainy season supports the link between measles dynamics and agricultural activity, upon which the country is economically heavily reliant (about 40% of its gross domestic product [36]).

The strong annual periodicity is consistent with previous findings [16,18,24]. The seasonal variation of the population density linked to the agricultural activity would be a strong driver of the seasonal measles transmission. The timing of the beginning of the measles season is consistent with the early phase of the annual agricultural labour migration, from mid-November to late April in a typical year [37]. The regularity of population movements associated with agricultural activity could provide an important window of opportunity for intervention. Seasonally timed targeted catch-up vaccination campaigns to reach migrating populations could be very powerful to break the annual pattern of outbreaks.

The second weaker periodicity illustrates the limits of the data needed to understand the chaotic component of measles dynamics in this area. Although weaker than the annual periodicity, this component remains substantial and characterizes an unpredictable aspect of measles transmission in Niger, which will be difficult to control programmatically. While we cannot attribute this periodicity to a specific mechanism with certainty, it partially reflects the building of pockets of susceptible hosts though birth, although it is not sufficient to explain the high temporal and spatial heterogeneity observed. Additional, more unpredictable, mechanisms such as spatial heterogeneities in vaccine coverage and measles reintroduction likely play important roles. No city in Niger exceeded the estimated critical community size for this setting, around 750 000 persons [18], which emphasizes the importance of annual measles reintroductions. However, the areas where the power spectrum ratio between the annual and 2–3-year periodicities favours the latter are likely to be more sensitive to reintroductions because of the lower contribution of the seasonally forced transmission there. In addition, the porosity of the borders between southern Niger and neighbouring countries with suboptimal coverage such as Nigeria ensures the mobility and mixing of infected and susceptible hosts on both sides [24]. To further explore this hypothesis we performed an additional analysis on the reported measles cases from 2005. Because of the occurrence of an outbreak response vaccination in 2004, the pool of susceptibles was mostly depleted and the cases notified in 2005 would mostly reflect introductions [24]. Districts with a higher proportion of their power spectrum attributed to the second periodicity for the year 2004 were more likely to have a higher attack rate than other districts in 2005 (electronic supplementary material, figure S14). This would support the hypothesis that reintroductions are at least partially responsible for this chaotic component. However, additional fine-scale data on the measles cases and movement patterns in the regional metapopulation, which spreads across national borders, would be necessary to further explore such underlying mechanisms.

Although a spatial hierarchy in the annual periodicity could not be observed as clearly as in England and Wales [6], some elements support its existence in Niger during our study period. Investigating the phase angles of the annual periodicity, we found some districts where outbreaks preceded neighbouring districts' outbreaks. Their phase shift varied slightly over time but remained mostly consistent. The spatial aggregation of the reported measles cases prevents us from investigating the spatial hierarchy in Niger at higher resolution. Our analysis can identify spatial hierarchy in the data at different periodicities, but it did not presume the identification of source districts. There could be some hierarchy among the districts we considered as potential sources. Additionally, we cannot discard the possibility that we observed only part of a wider transnational metapopulation with some of the source districts outside of Niger.

The significant association with the rainy season likely reflects the impact of shifting human activities. It is consistent with what is expected in countries with agriculturally driven economies: fluctuating population densities as seasonal agricultural labourers transition between urban and rural areas, creating a strong seasonal forcing in transmission [15,22]. This is another facet of the phenomenon captured by the strong regularity of the annual periodicity and further supports the theory that an intervention focusing on this mobile population could strongly impact measles transmission in Niger.

Our findings are consistent with current literature on measles dynamics in Niger. Anthropogenic nightlights demonstrate a decrease in population density during rainy seasons in Niamey, Zinder and Maradi during the same study period. Furthermore, explicitly incorporating this seasonal variation of population size into a susceptible–exposed–infected–recovered (SEIR) model provided correct predictions on the timing and the amplitude of a 2003–2004 measles outbreak in Niamey for administrative divisions within the city [16]. This supports our interpretation of the potential mechanism driving the yearly periodicity. In addition, the potential role of reintroductions in measles dynamics in Niger has previously been explored, and it highlighted the strong connectivity of Niger with Nigeria [24]. It is likely that the second periodicity is driven by a complex association of mechanisms, but reintroductions in this context are probably part of them.

This analysis has some limitations. Reported measles cases are aggregated at the district level, and this necessarily implies variable spatial resolution. In addition, there is likely to be a differential reporting bias between districts with a lower probability of case reporting in rural areas where the population is sparse and fragmented across space and where the health system is also likely weaker [38]. However, those areas are also less densely populated and less likely to have large outbreaks. This makes the direction of this bias hard to assess, but the reporting bias probably decreased the statistical power to detect the periodicities and the environmental associations in those areas with a satisfying precision. However, this is a common issue with surveillance data in low-income countries and it emphasizes the need for structurally improving the surveillance system.

The band used to explore the second periodicity is too narrow to capture all the variety of the GWPS in this area (electronic supplementary material, figure S8). However, shifting the band to slightly higher or lower values would encounter a similar issue. Furthermore, this highlights the variety of situations displayed by the districts across Niger and the potential complexity of the mechanisms involved in this second periodicity. The existence of a second periodicity relying on other transmission mechanisms remains, and it will become more important to tackle as vaccination coverage increases.

The reporting bias could influence the analysis of the reintroductions in 2005. The districts with lower population density tend to be less connected, potentially having fewer reintroductions, but also being less likely to report them than more densely populated districts. There is no statistical solution to resolve this potential bias with the data currently available.

The pattern displayed by HC based on the average phase coherency for the annual periodicity (figure 3) was relatively robust to slight variations in the number of clusters considered. Increasing the number of clusters did not modify the interpretation despite the added complexity in the spatial pattern (electronic supplementary material, figure S12).

Our analysis presents several strengths and original findings. We provide important hindsight on spatial transmission dynamics regarding patterns that are still relevant and actionable today. Because the estimated vaccine coverage is higher today in Niger, 77% for one dose and 48% for two doses at the country level in 2018 [14], such patterns might be harder to detect with more recent data, but the main trends in measles dynamics are still driven by similar underlying factors. Aggregation patterns observed from 1995 to 2004 [16] continue to be observed as recently as 2013–2018 (data not published). Agriculture continues to be a major economic activity in Niger, and its dependence on environmental drivers and its impact on population movement remains. Similarly, the borders are still porous and the transnational movement of infected and susceptible individuals continues [23,39,40]. Vaccine coverage data at high spatial resolution are not available for Niger but it is likely that the suboptimal coverage between 1995 and 2004 was already heterogeneous. Although vaccine coverage increased during the last decade [14], inequalities in healthcare remain [41]. Spatial heterogeneity in vaccine coverage persists and is a source of chaos in measles dynamics in Niger today, where 5034 measles cases were reported in 2018 [42].

## 5. Conclusion

The dual dynamics we detected in reported measles cases in Niger from 1995 to 2005 are likely to be due to mechanisms specific to this setting, with such an economic agricultural driver. Applying solutions designed for high-income countries has already proven to be insufficient to control measles in this setting. Tailoring targeted interventions to plan catch-up vaccination efforts for migrating populations could help break the very strong annual measles seasonality. However, interrupting the more chaotic component of the measles dynamic would require more challenging and complex interventions. A large regional improvement in access to care and reducing health inequalities would reduce spatial heterogeneities in vaccination coverage. Reducing measles reintroductions can only be done if vaccine coverage increases across the metapopulation. Despite a substantial decrease in the under-5 mortality rate (U5MR) achieved by the Millennium Development Goals [3], sub-Saharan Africa remains far from achieving the sustainable development goal (SDG) of ending preventable deaths of newborns and children under 5 years of age and reaching a U5MR as low as 25 per 1000 live births by 2030 [43]. Measles is no longer the main cause of deaths in children under 5 years in this region, but its mortality and morbidity remain high. Additionally, measles-related mortality is likely to be underestimated with respect to the downstream consequences of immune amnesia [44]. Adapting vaccination strategies for this setting is then necessary to break its transmission. Furthermore, such an achievement would also benefit the management of other preventable diseases and put additional SDG goals within reach.

## Supplementary Material

Supplementary material

## Supplementary Material

Epidemic curves of weekly reported measles cases at the district level from 1995 to 2004, Niger

## Supplementary Material

Local wavelet power spectrum of the reported measles cases at district level from 1995 to 2004 for 20 districts in Niger.

## Supplementary Material

Local wavelet power spectrum of the reported measles cases at district level from 1995 to 2004 for 18 districts in Niger.

## Supplementary Material

Evolution of the phase angle of the annual periodicity in every district in Niger, 1995-2004

## Data accessibility

The data and the code are available from the Dryad Digital Repository: https://doi.org/10.5061/dryad.1jwstqjrd [45].
