# Supplementary material for: Investigating persistent measles dynamics in Niger and associations with rainfall
Source: J R Soc Interface. 2020 Aug 26;17(169):20200480. doi: 10.1098/rsif.2020.0480 (PMC7482562; doi:10.1098/rsif.2020.0480)
Supplement: Supplementary material [file rsif20200480supp1.pdf]

Supplementary material

Defining the rainy season

The following Figure illustrates how the rainy season was defined using Maradi, Niamey, and Zinder districts as examples.

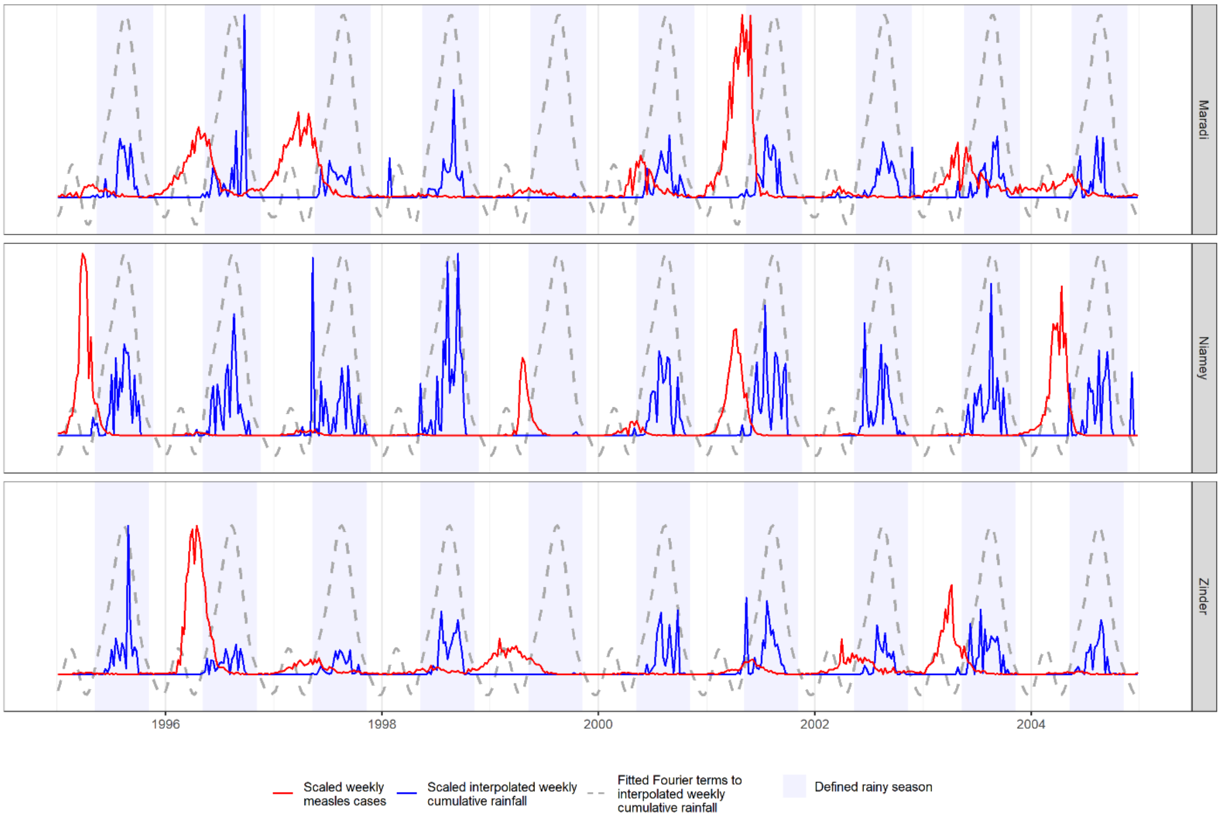

Figure 7: Scaled weekly reported cases (red), scaled average interpolated weekly cumulative rainfall (blue), Fourier terms fitted to the average interpolated weekly cumulative rainfall (dashed grey), and defined rainy season (pale blue rectangles).

The red and blue lines are respectively the weekly reported measles cases and the interpolated cumulative weekly rainfall averaged at district level (scaled to be able to visualize them with the fitted Fourier terms with similar value ranges). Because the timing of the rainy season likely mattered more than the actual value of the weekly cumulative rainfall in triggering seasonal labor migration, and influencing measles transmission, we defined the rainy season based on the seasonal trend observed. We fitted Fourier terms to the interpolated weekly cumulative rainfall, the grey dashed line, and used it to define the rainy season, the pale blue area. The procedure was done for every district separately.

Power spectra at district level

Local power spectra

The local power spectra (see Electronic Supplementary Material) were obtained after wavelet decomposition using the Morlet wavelet after square root transformation of the time series. The dashed and dotted contour lines delineate areas with a p-value below 0.05 and below 0.001 respectively. The p-value contour lines were estimated using permutation tests (1000 permutations). The gray shaded area is the cone of influence, indicating areas potentially influenced by an edge effect.

Global power spectra

The global power spectra (Figure 8) were calculated by averaging the local power spectra over the 10-year period of the data. The dashed and dotted lines show the 95% and 99.9% confidence intervals. They were calculated using permutation tests (1000 permutations). A dominant periodicity around 1 year, and a second periodicity peaking around 2-3.5 years depending on the district are statistically significant.

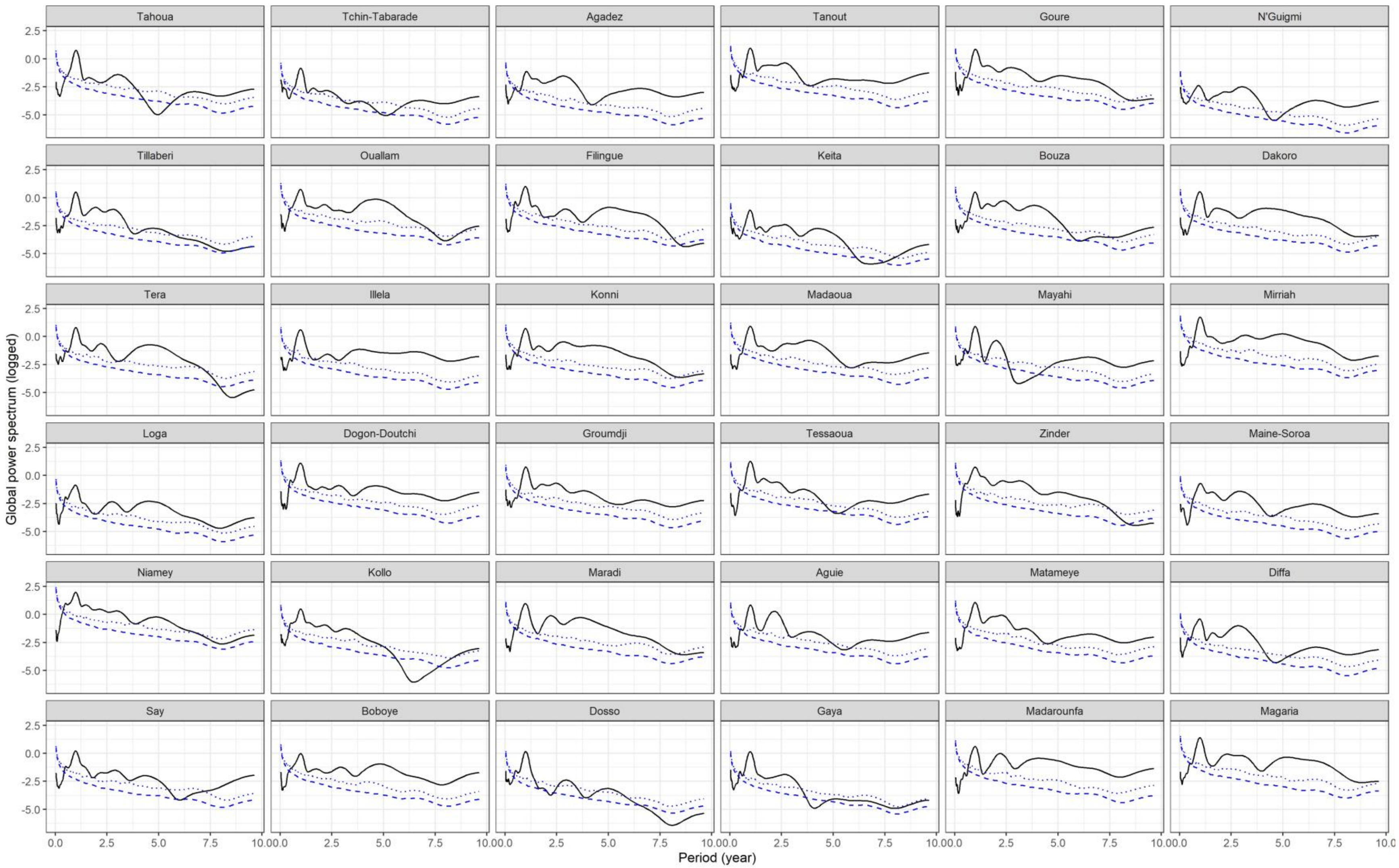

Figure 8: Global wavelet power spectra (GWPS) with 95% and 99.9% boundaries per district. The boundaries were obtained after calculating the GWPS of 1000 random permutations of the original time series and estimating the 95th and 99.9th percentiles among the permutations at every value of the period.

Phase shift block by block

Periodicity of 2-3 years

The following figure displays a map of the blocks identified when considering the eight districts with a phase shift that leads the others when analyzing the periodicity of 2-3 years, in A, and the calculated phase shifts of the districts in blocks with at least one district potentially following, in B.

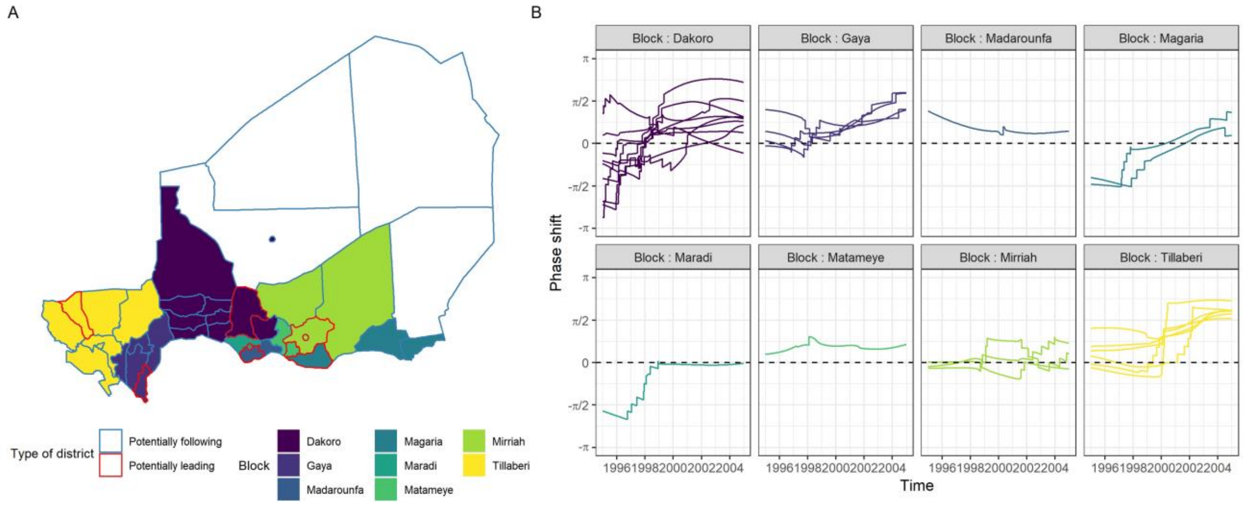

Figure 9: Map of the blocks grouping potential source and potential sink districts based on the distance between population weighted centroids (A) and phase shifts of the potential sink districts using the closest potential source district* (B) considering the 2-3 years periodicity in reported measles cases from 1995 to 2004 in Niger

Annual periodicity

The following figure displays the identified blocks, in A and C, and the phase shifts of the districts in the blocks with at least one district that was not among those considered to be ahead of the others when analyzing the annual periodicity, in B and D. A and B show the situation when considering the five districts ahead of the other districts as the ones potentially leading, whereas C and D consider the ten districts ahead of the other districts.

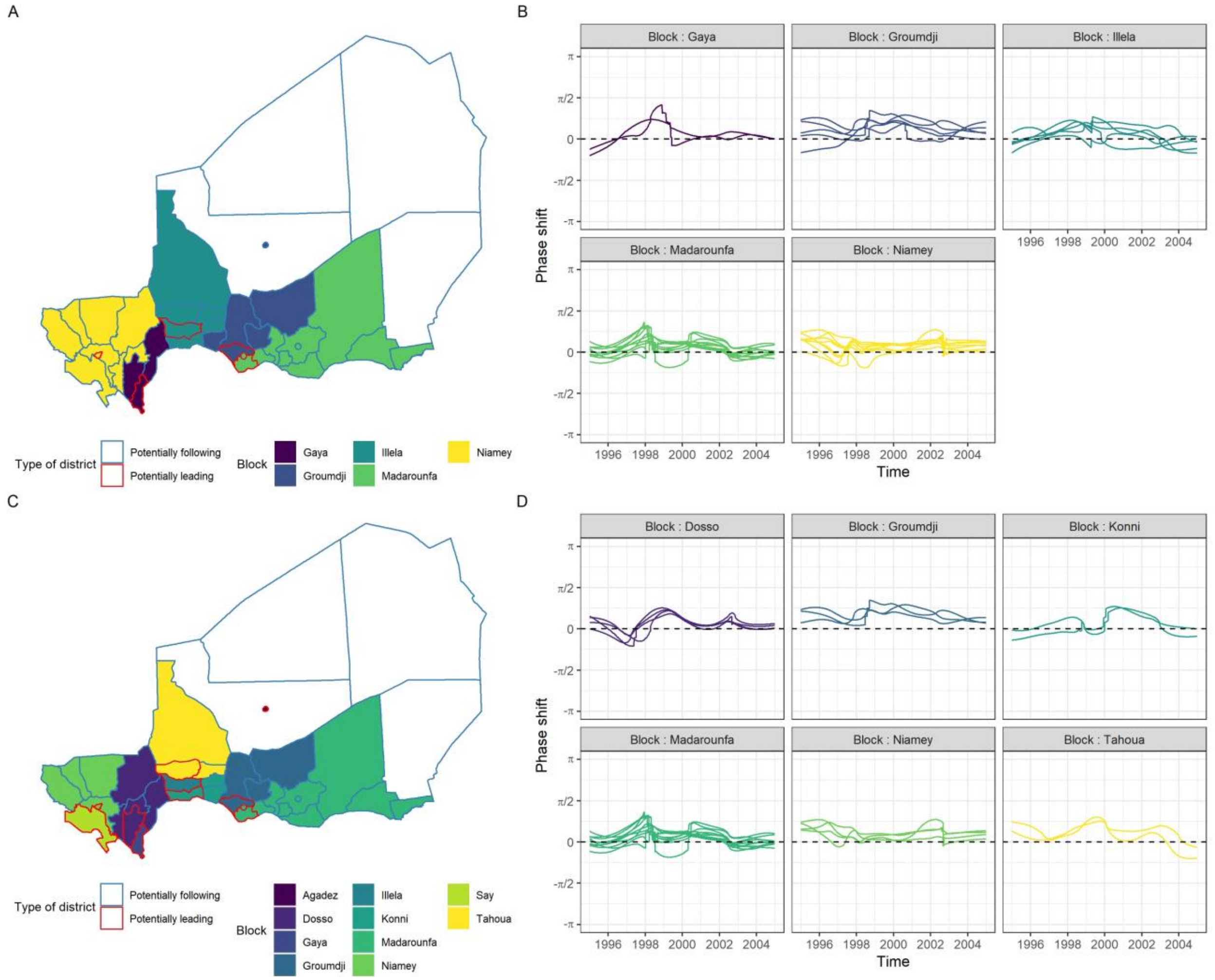

Figure 10: Map of the blocks grouping potential source and potential sink districts based on the distance between population weighted centroids (A and C) and phase shifts of the potential sink districts using the closest potential source district* (B and D) considering the annual periodicity in reported measles cases when considering five (A and B) or ten (C and D) potential source districts from 1995 to 2004 in Niger

*: Potential source districts with no potential sink district are not represented in B and D

Detail of the adjusted risk ratio of the rainy season and measles incidence

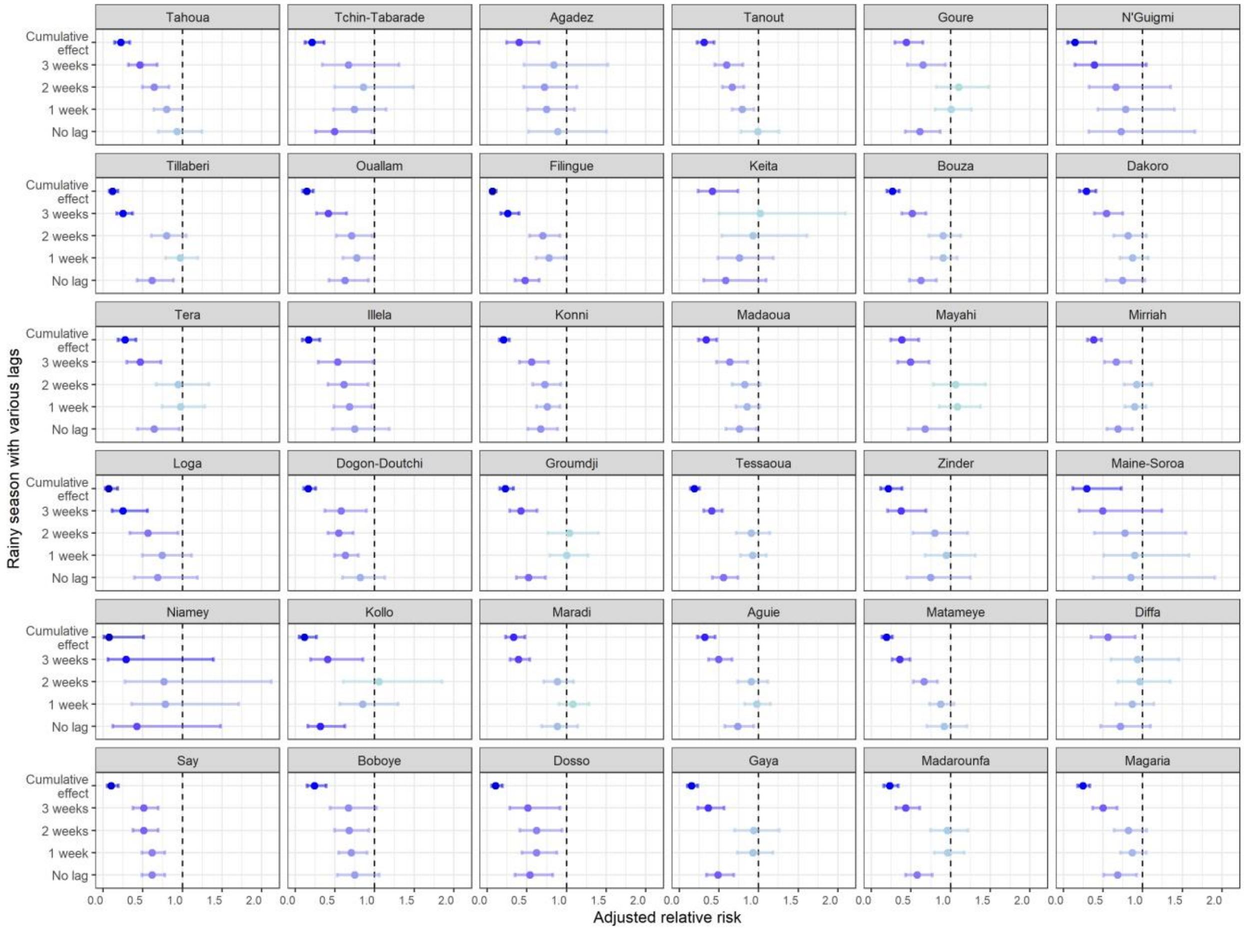

Figure 11: Adjusted risk ratio of the association between the rainy season and measles incidence with 0 to 3 weeks lag and the cumulative effect and their 95% confidence interval at district level* from 1995 to 2004 in Niger.
*: The districts are organized North to South from top to bottom and West to East from left to right.

The facets of the different districts are organized top to bottom from North to South and left to right from West to East.

Check of the residuals of the quasi-Poisson regression models

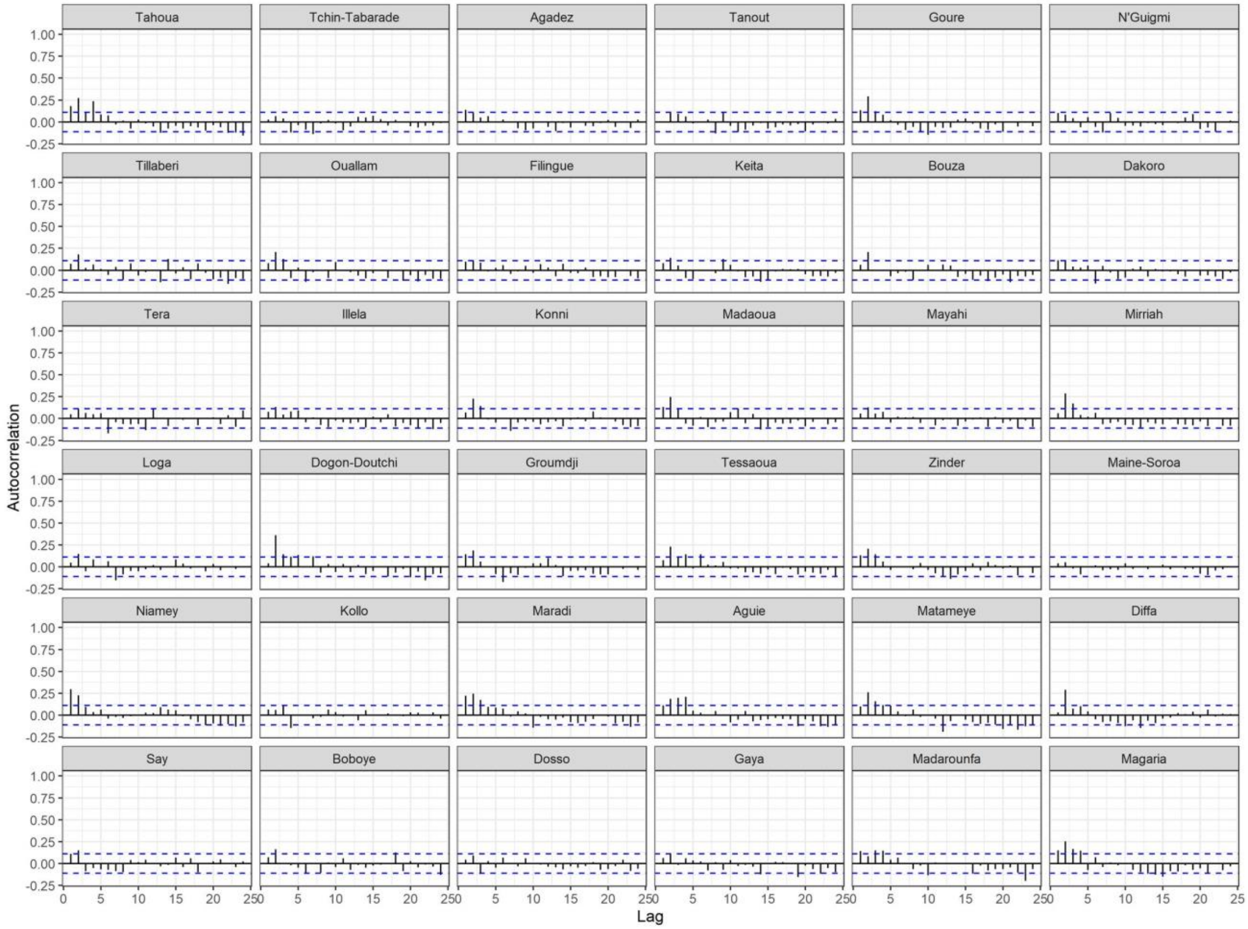

Figure 12: Autocorrelation in the residuals of the models providing the estimates of the adjusted odds ratios of the association between the rainy season and measles incidence interval at district level* from 1995 to 2004 in Niger.
*: The districts are organized North to South from top to bottom and West to East from left to right.

Little to no autocorrelation remained in the residuals of the models presented.

Sensitivity of the occurrence of the rainy season to the spatial range used to average rainfall and its impact on its adjusted risk ratio

Some of the seasonal movements correspond to people leaving urban areas to work in agricultural areas, which are less densely populated. Their movement can be influenced to some extent by the weather in areas surrounding the urban areas, due to its impact on agriculture. Determining the exact spatial range we should consider to average rainfall and temperature is impossible.

We repeated the analysis considering two alternative approaches regarding rainfall:

- Averaging the interpolated rainfall over districts and their contiguous neighbors

- Averaging the interpolated rainfall over the area centered on the population weighted centroid of the districts with a radius corresponding to the maximum distance covered on foot. The radius was approximately 112 km, considering the distance covered walking 8 hours/day and 5 days/week in Niger (1).

We then dichotomized the occurrence of the rainy season using the approach described previously (see Defining the rainy season). Figure 13 below highlights that the two alternative approaches (in red and blue) lead to very similar timings of the occurrence of the rainy season and comparable interpretation to the one presented in the manuscript (in green).

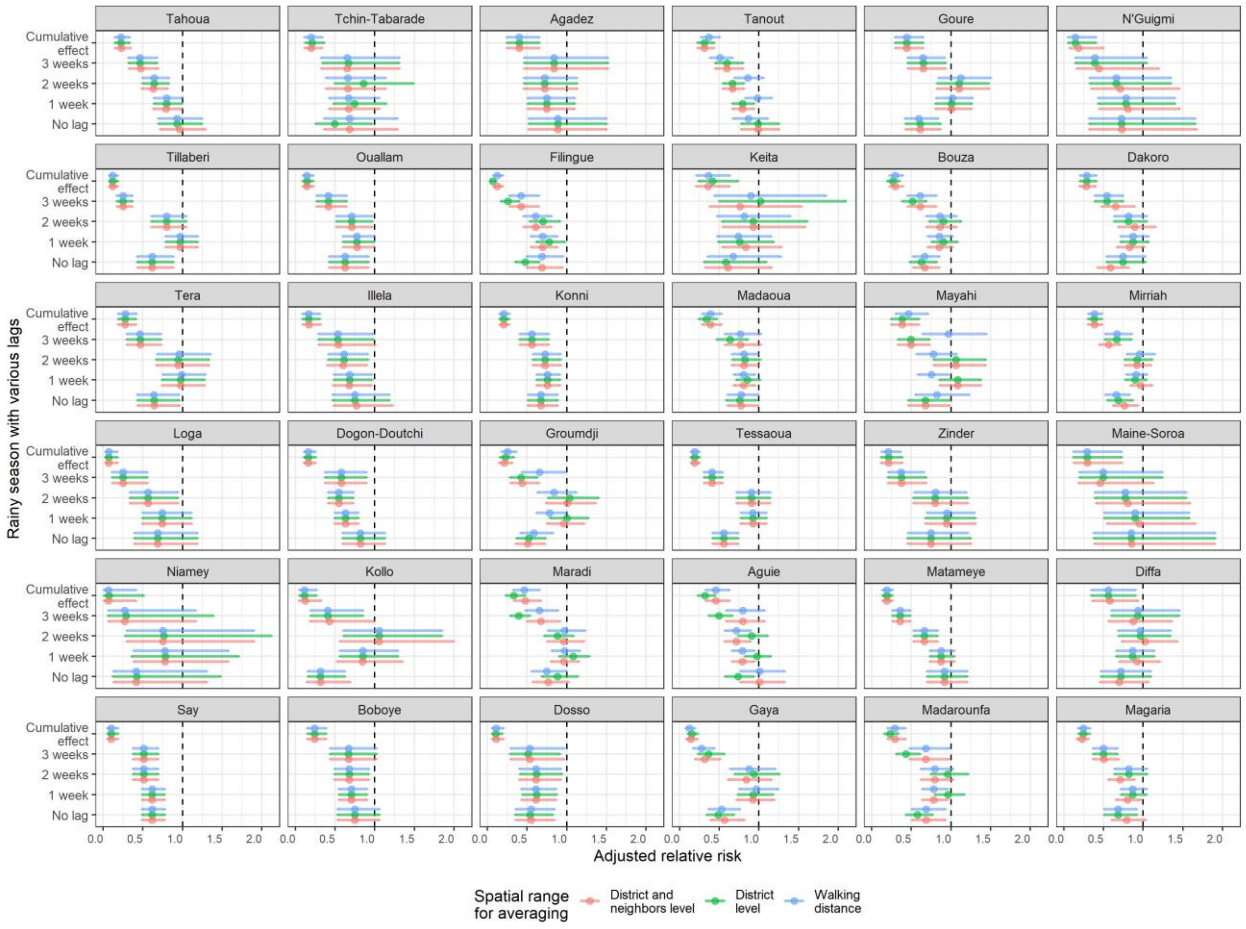

Figure 13: Impact of defining the occurrence of the rainy season after averaging the rainfall over different spatial ranges on its adjusted relative risk.

Clustering

In addition to guiding our choice of the number of clusters using the Elbow (2) and silhouette plots (3), and the gap statistic (4), we explored the impact of choosing a different number of clusters on the spatial pattern observed.

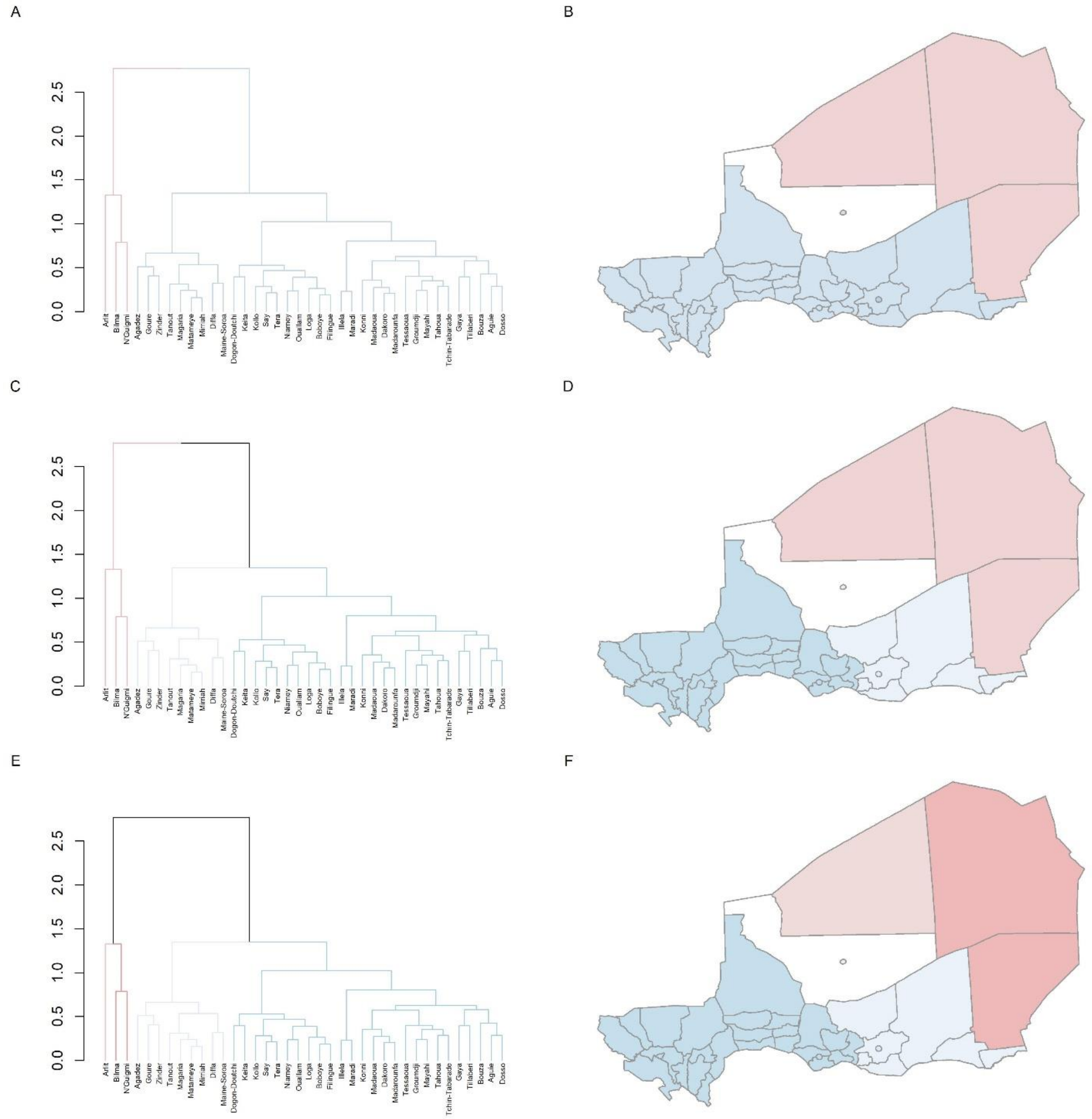

Figure 14: Clustering tree (A, C, and E) and mapping (B, D, and F) of the clustering based on average cophenetic correlation for the annual periodicity when considering two (A and B), three (C and D), or four (E and F) clusters from 1995 to 2004 in Niger

Figure 14 presents in A and B the clustering tree and the resulting mapped clustering based on the average phase coherency considering the annual periodicity as presented in the manuscript, with two clusters. The following figures C and D, and then E and F, are the same figures but considering respectively three and four clusters. As the number of clusters considered increases, the spatial pattern is still consistent with two areas following two very different dynamics: the north and the south of the country. However, they emphasize subdivisions within the two clusters presented where the similarity is even stronger following a west/east axis. Those areas appearing with the increasing number of clusters include contiguous districts. Choosing a cutoff on the clustering tree leading to two, three, or four clusters does not fundamentally change the interpretation of the observed pattern. Whether the

boundaries of those additional clusters can be explained by road connectivity and/or the spatial distribution of ethnicity is unclear.

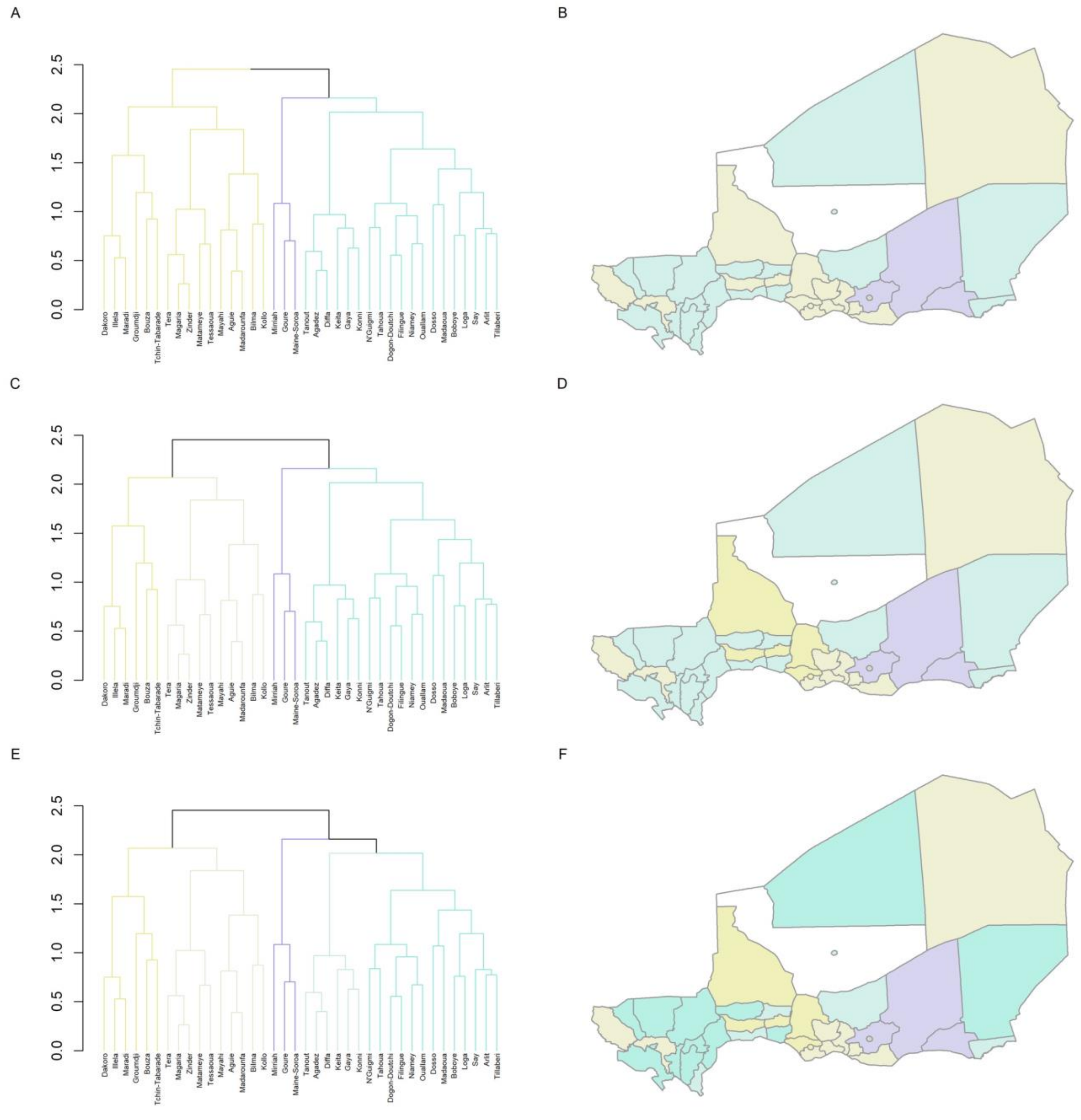

Figure 15: Dendrogram (A, C, and E) and mapping (B, D, and F) of the clustering based on average cophenetic correlation for the 2-3.5 years periodicity when considering three (A and B), four (C and D), or five(E and F) clusters from 1995 to 2004 in Niger

Figure 15 presents in A and B the clustering tree and the resulting mapped clustering based on the average phase coherency considering the 2-3 years periodicity as presented in the manuscript, with three clusters. The following figures C and D, and then E and F, are the same figures but considering respectively four and five clusters. Except for the cluster including Mirriah, Goure, and Maine-Soroa (purple in Figure 14), further splitting the clusters reveals areas also geographically fragmented. Again, considering three, four, or five clusters is still consistent with a mostly erratic pattern.

Overdispersion parameters

Table 1: Significance for an overdispersion higher than 1, and estimates of the overdispersion parameters of the final quasi-poisson models

District	p-value[a]	Overdispersion parameter
Agadez	p<0.001	5.04
Aguie	p<0.001	6.71
Boboye	p<0.001	4.49
Bouza	p<0.001	6.90
Dakoro	p<0.001	6.84
Diffa	p<0.001	5.94
Dogon-Doutchi	p<0.001	10.44
Dosso	p<0.001	6.06
Filingue	p<0.001	7.36
Gaya	p<0.001	5.65
Goure	p<0.001	10.49
Groumdji	p<0.001	9.40
Illela	p<0.05	11.93
Keita	p<0.001	6.89
Kollo	p<0.001	16.28
Konni	p<0.001	8.91
Loga	p<0.05	3.81
Madaoua	p<0.001	8.57
Madarounfa	p<0.001	4.02
Magaria	p<0.001	13.92
Maine-Soroa	0.07	11.96
Maradi	p<0.001	8.98
Matameye	p<0.001	8.23
Mayahi	p<0.001	8.98
Mirriah	p<0.001	13.23
N'Guigmi	p<0.001	5.54
Niamey	0.06	231.92
Ouallam	p<0.001	10.16
Say	p<0.001	5.15
Tahoua	p<0.001	6.40
Tanout	p<0.001	10.57
Tchin-Tabarade	p<0.001	8.55
Tera	p<0.001	10.88
Tessaoua	p<0.001	10.51
Tillaberi	p<0.001	6.28
Zinder	p<0.05	13.65

a: The null hypothesis for the test performed is that the overdispersion parameter is 1 using Poisson regression

The overdispersion in the models was tested using the approach described by Cameron and Trivedi (5) testing if the overdispersion was different from 1 using similar models but with Poisson regression. The Table 1 provides the p-value of the tests and also the estimate of the overdispersion parameter in the final quasi-Poisson models.

Measles reintroductions in 2005

Because of the supplementary immunization activities occurring in 2004, the pool of susceptible was mostly depleted the following year (6). A high level of population immunity would highlight measles re-introductions among the reported cases by removing the cases attributable to local transmission. We calculated the attack rate (AR) for every district for the year 2005 to account for the population size. More populated district would be more likely to receive travelers and also be more likely to receive measles introductions. We also calculated the proportion of the power spectrum of the year 2004 due to the second periodicity, estimated through wavelet analysis for the period 1995-2004. We focused on the year preceding the re-introductions because of the variation over time of the power spectrum of specific periodicity. We then log-transformed the AR of each district and looked for an association with the proportion of the 2004 power spectrum attributable to the second periodicity by linear regression (adjusted R^2=0.11) after excluding an outlier. Bilma was identified as the only outlier with a Cook's distance of 8.25. This district had the lowest population size and its AR could be sensitive to small variation of reported cases. There was a significant positive association (p=0.025) (Figure 16) supporting the premise that districts with a higher proportion of their power spectrum in the year 2004 due to the second periodicity had higher AR in 2005. The association remained significant with the inclusion of the outlier (p=0.021).

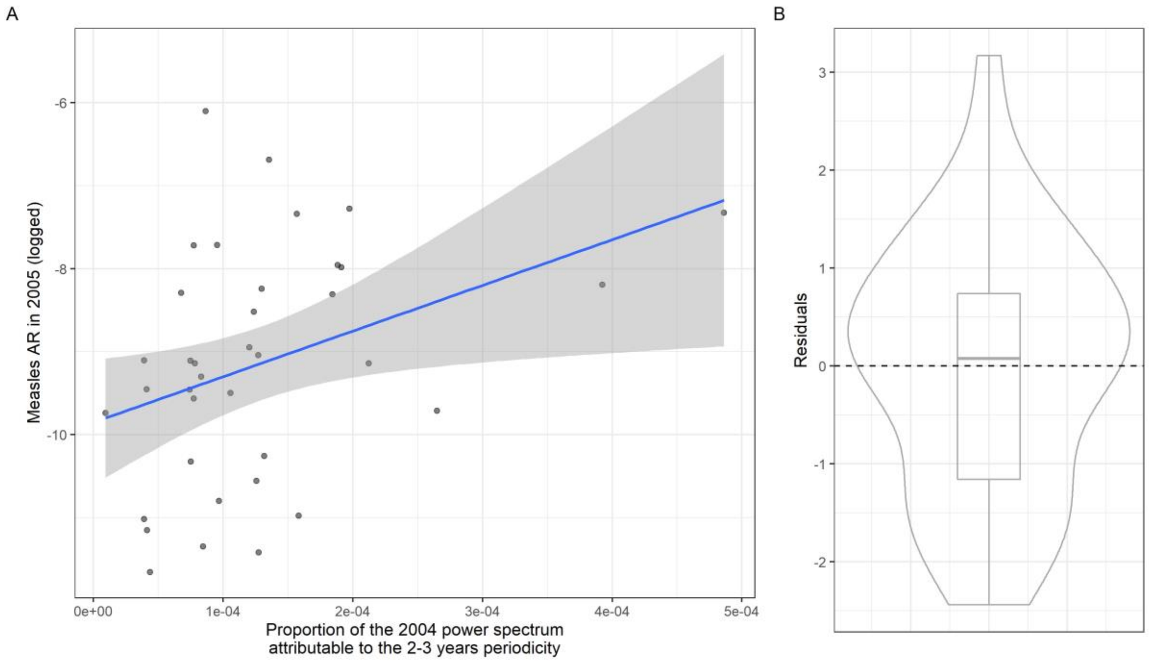

Figure 16: Scatter plot of the proportion of the 2004 spectrum attributable the 2-3 years periodicity against logged measles attack rate (AR) with a regression line(A) and a boxplot of residuals of the regression line (B)

To account for a potential reporting bias, with more reliable data in more populated areas, we reran the linear regression with weights corresponding to population sizes. The positive association still held and remained significant (p=0.022).

References

1. Blanford JI, Kumar S, Luo W, MacEachren AM. It's a long, long walk: accessibility to hospitals, maternity and integrated health centers in Niger. Int J Health Geogr. 2012 Jun 27;11:24.

2. Ketchen DJ, Shook CL. The Application of Cluster Analysis in Strategic Management Research: An Analysis and Critique. Strateg Manag J. 1996;17(6):441–58.

3. Rousseeuw PJ. Silhouettes: A graphical aid to the interpretation and validation of cluster analysis. J Comput Appl Math. 1987 Nov 1;20:53–65.

4. Tibshirani R, Walther G, Hastie T. Estimating the number of clusters in a data set via the gap statistic. J R Stat Soc Ser B Stat Methodol. 2001;63(2):411–23.

5. Cameron AC, Trivedi PK. Regression-based tests for overdispersion in the Poisson model. J Econom. 1990 Dec 1;46(3):347–64.

6. Bharti N, Djibo A, Ferrari MJ, Grais RF, Tatem AJ, McCabe CA, et al. Measles hotspots and epidemiological connectivity. Epidemiol Infect. 2010 Sep;138(9):1308–16.
