# Supplementary figures and images for: Investigating persistent measles dynamics in Niger and associations with rainfall
Source: J R Soc Interface. 2020 Aug 26;17(169):20200480. doi: 10.1098/rsif.2020.0480 (PMC7482562; doi:10.1098/rsif.2020.0480)

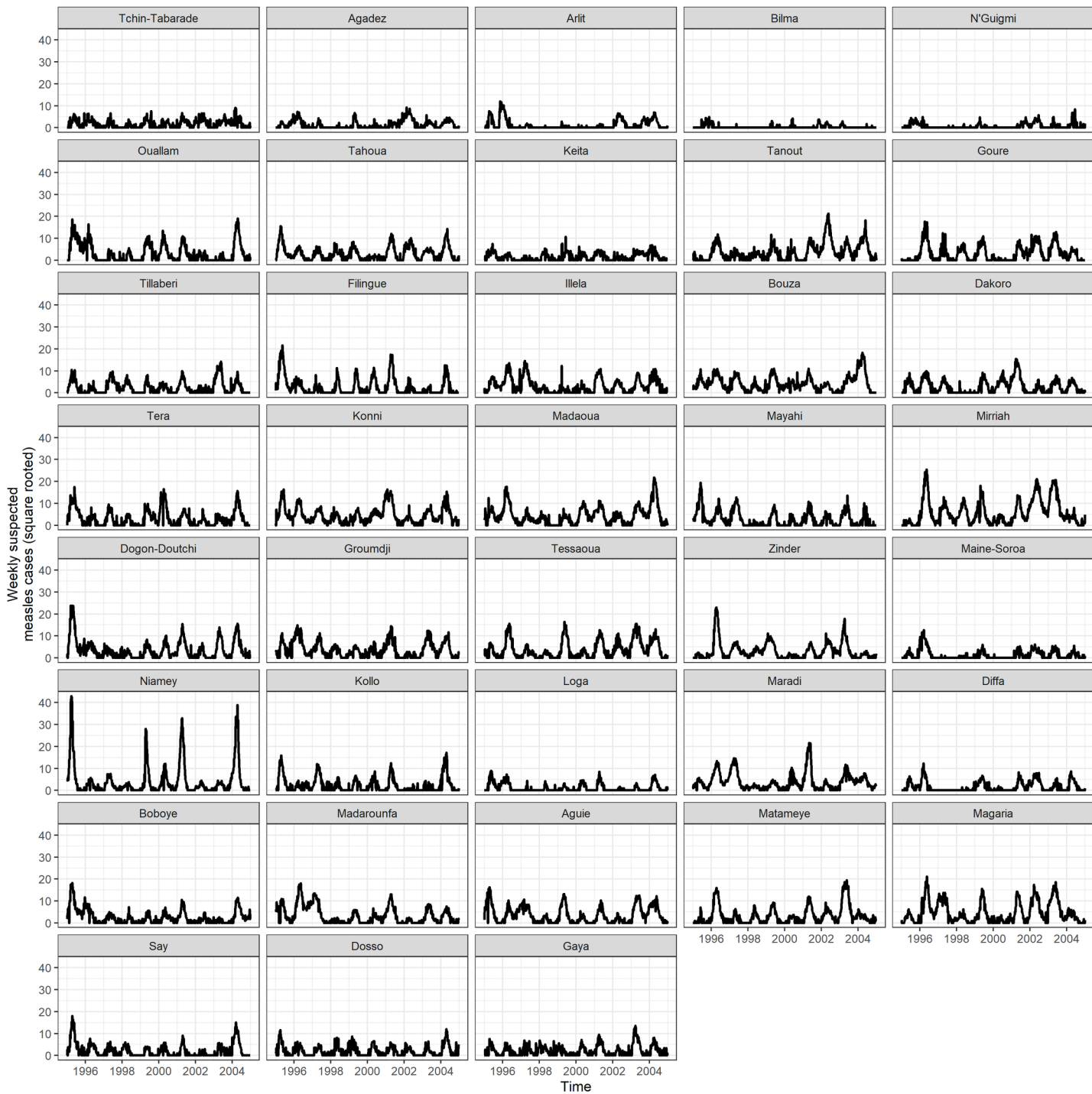

Supplement: Epidemic curves of weekly reported measles cases at the district level from 1995 to 2004, Niger [file rsif20200480supp2.pdf]

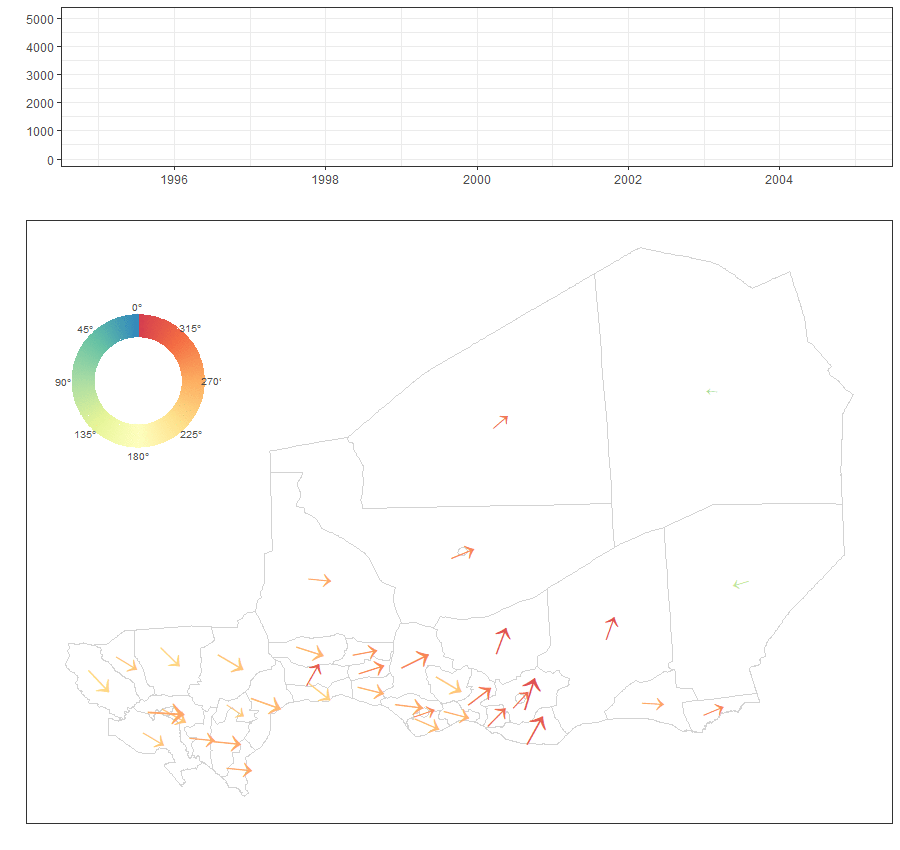

Supplement: Evolution of the phase angle of the annual periodicity in every district in Niger, 1995-2004 [file rsif20200480supp5.gif]
