# Supplementary material for: Investigating persistent measles dynamics in Niger and associations with rainfall
Source: J R Soc Interface. 2020 Aug 26;17(169):20200480. doi: 10.1098/rsif.2020.0480 (PMC7482562; doi:10.1098/rsif.2020.0480)
Supplement: Local wavelet power spectrum of the reported measles cases at district level from 1995 to 2004 for 20 districts in Niger. [file rsif20200480supp3.pdf]

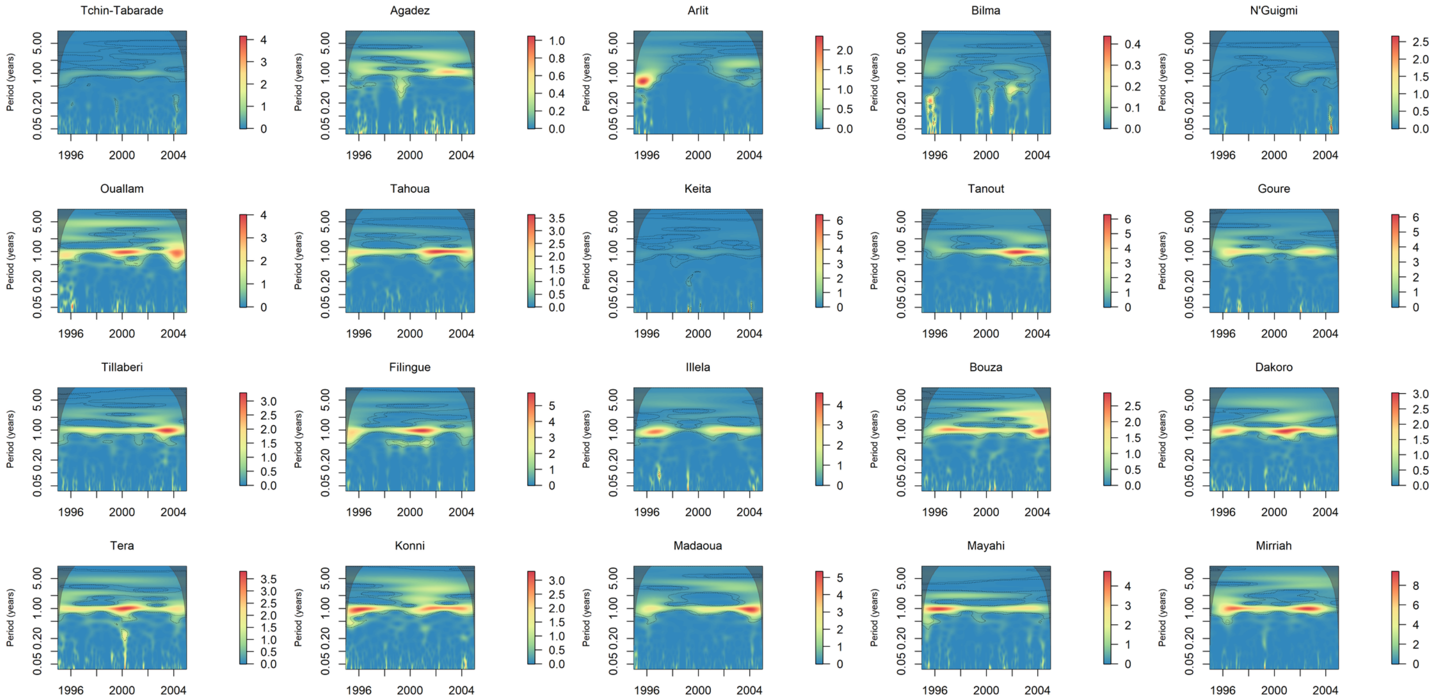
Tchin-Tabarade
Agadez
Arlit
Bilma
N'Guigmi
Ouallam
Tahoua
Keita
Tanout
Goure
Tillaberi
Filingue
Illela
Bouza
Dakoro
Tera
Konni
Madaoua
Mayahi
Mirriah
Period (years)
0.05 0.20 1.00 5.00
1996 2000 2004
