# Supplementary material for: Investigating persistent measles dynamics in Niger and associations with rainfall
Source: J R Soc Interface. 2020 Aug 26;17(169):20200480. doi: 10.1098/rsif.2020.0480 (PMC7482562; doi:10.1098/rsif.2020.0480)
Supplement: Local wavelet power spectrum of the reported measles cases at district level from 1995 to 2004 for 18 districts in Niger. [file rsif20200480supp4.pdf]

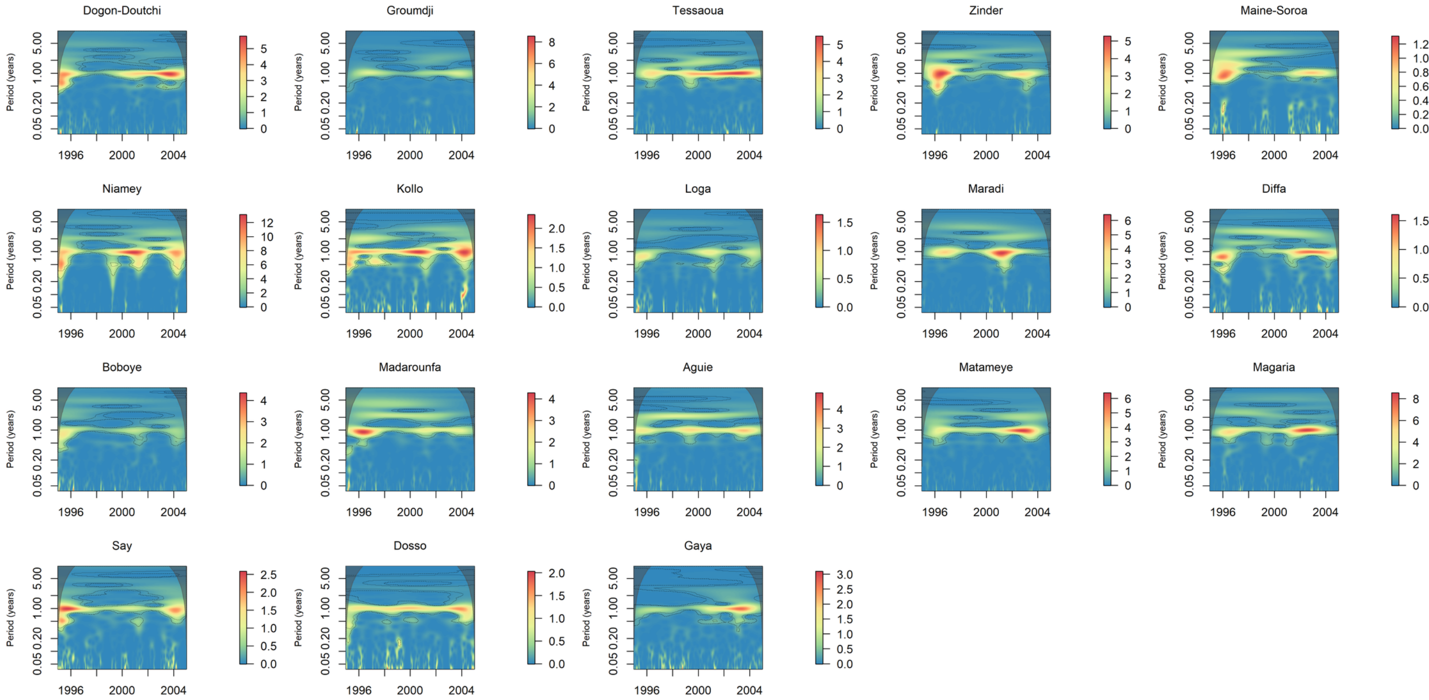
Dogon-Doutchi
Groumdji
Tessaoua
Zinder
Maine-Soroa
Niamey
Kollo
Loga
Maradi
Diffa
Boboye
Madarounfa
Aguie
Matameye
Magaria
Say
Dosso
Gaya
Period (years)
0.05 0.20 1.00 5.00
1996 2000 2004
